# Bi-Exponential 3D UTE-T1ρ Relaxation Mapping of Ex Vivo Human Knee Patellar Tendon at 3T

**DOI:** 10.3390/bioengineering11010066

**Published:** 2024-01-09

**Authors:** Bhavsimran Singh Malhi, Dina Moazamian, Soo Hyun Shin, Jiyo S. Athertya, Livia Silva, Saeed Jerban, Hyungseok Jang, Eric Chang, Yajun Ma, Michael Carl, Jiang Du

**Affiliations:** 1 Department of Radiology, University of California, La Jolla, San Diego, CA 92037, USA; bmalhi@health.ucsd.edu (B.S.M.); dmoazamian@health.ucsd.edu (D.M.); shs033@health.ucsd.edu (S.H.S.); jathertya@health.ucsd.edu (J.S.A.); ltsilva@ucsd.edu (L.S.); sjerban@health.ucsd.edu (S.J.); h4jang@health.ucsd.edu (H.J.); e8chang@health.ucsd.edu (E.C.); yam013@health.ucsd.edu (Y.M.); michael.carl@ge.com (M.C.); 2 Radiology Service, Veterans Affairs San Diego Healthcare System, La Jolla, San Diego, CA 92161, USA; 3 General Electric Health Care, San Diego, CA 92037, USA

**Keywords:** ultrashort echo time, MRI, patellar tendon, bicomponent

## Abstract

**Introduction**: The objective of this study was to assess the bi-exponential relaxation times and fractions of the short and long components of the human patellar tendon ex vivo using three-dimensional ultrashort echo time T1ρ (3D UTE-T1ρ) imaging. **Materials and Methods**: Five cadaveric human knee specimens were scanned using a 3D UTE-T1ρ imaging sequence on a 3T MR scanner. A series of 3D UTE-T1ρ images were acquired and fitted using single-component and bi-component models. Single-component exponential fitting was performed to measure the UTE-T1ρ value of the patellar tendon. Bi-component analysis was performed to measure the short and long UTE-T1ρ values and fractions. **Results**: The single-component analysis showed a mean single-component UTE-T1ρ value of 8.4 ± 1.7 ms for the five knee patellar tendon samples. Improved fitting was achieved with bi-component analysis, which showed a mean short UTE-T1ρ value of 5.5 ± 0.8 ms with a fraction of 77.6 ± 4.8%, and a mean long UTE-T1ρ value of 27.4 ± 3.8 ms with a fraction of 22.4 ± 4.8%. **Conclusion**: The 3D UTE-T1ρ sequence can detect the single- and bi-exponential decay in the patellar tendon. Bi-component fitting was superior to single-component fitting.

## 1. Introduction

Tendon disease is one of the most common causes for patients to seek medical attention from their general practitioner, accounting for 30% of musculoskeletal consultations [1]. Tendon pathology is not limited to a specific body area, but can also affect multiple joints, such as the shoulder, elbow, hand, knee, and ankle. This condition can impact individuals of all ages, ranging from young athletes to senior citizens.

Tendinopathy can be defined as pain or loss of function in a tendon and is a blanket term for ‘tendinitis’, ‘tendinosis’, and ‘tenosynovitis’, which are used to define inflammatory and degenerative changes in the tendons [2,3]. Patellar tendinopathy is common in sports that involve jumping, such as basketball and volleyball, as well as in sports that require quick changes in direction and acceleration or deceleration [4,5,6]. The prevalence of this condition varies from around 8.5% in non-elite athletes to as high as 50% in elite athletes [7,8]. Numerous theories have been put forward regarding the etiology of tendinopathy, with multiple studies demonstrating associations between tendinopathy and elevated levels of proteoglycans, as well as disruptions in the collagen network [9,10,11,12].

Magnetic resonance imaging (MRI) is considered the gold standard for imaging tendons, providing excellent soft tissue contrast [13]. Quantitative MRI of tendons has been performed using T1, T2*, and more recently, T1ρ sequences. T1ρ relaxation time is fully named spin-lattice relaxation time in the rotating frame and provides extra information on tissues beyond the T1 and T2 weighted contrast. Spin-spin or T2 relaxation involves a dephasing procedure in the transverse plane in which the transverse magnetization decays to zero exponentially. If an external RF pulse, called a spin-lock pulse, is applied with the same precession frequency as spins in the transverse plane, the spins undergo a slower relaxation process known as T1ρ relaxation [14,15]. T1ρ is sensitive to low-frequency macromolecular motions (100 Hz to a few kHz) around or at the spin-lock frequency and is of particular interest in musculoskeletal imaging [15,16,17]. The T1ρ relaxation time is capable of measuring the alterations in biochemical properties related to proteoglycans (PG), water concentration, as well as the impairment of the collagen network and its anisotropy [15].

Highly organized tissues like tendons and ligaments have T2 relaxation times in the order of a few milliseconds. The lack of visualization of these structures using conventional sequences hampers their ability to detect early tendon degeneration with sufficient sensitivity. Ultrashort echo time (UTE) pulse sequences, on the other hand, have TEs in the range of 0.05–0.20 milliseconds and can acquire signals from these tissues with a majority of short T2 components, which could be helpful in the early diagnosis of diseases [18]. The T1ρ relaxation time can help to quantify the changes. Meanwhile, biological tissues have demonstrated distinct water compartments (or pools) using T2, T2*, and, more recently, T1ρ relaxation times [19,20,21,22]. Multiple studies have shown that tendons have two distinct water components: bound water and free water [22,23,24,25,26,27,28]. Free water is located between the network of collagen bundles, and bound water is closely associated with collagen and/or peptidoglycan [22,27]. Several studies have shown that the short or fast relaxation component correlates with tendon pathology and clinical scores [22,24,27,28]. Single exponential models are incapable of differentiating between short and long relaxation components. However, techniques for mapping bi-component T1ρ have the potential to enhance the sensitivity and specificity of T1ρ analysis by evaluating individual water components of the tendon.

Tendons consist mainly of collagen, specifically type 1, which accounts for approximately 60–85% of their dry weight. Approximately 1–5% of the dry weight of tendons is composed of proteoglycans, while elastin contributes up to 2% of their weight [29,30]. The short component is thought to be related to water which is closely associated with collagen, and the long component is related to free water. Peto et al. utilized T2* analysis to reveal four distinct water components, as evidenced by their demonstration of four T2* components [31].

There is a limited amount of research on the analysis of bi-component T1ρ in tissues. Tissues such as cartilage, skeletal muscle, and brain, which have relatively long T2 values, have been examined using conventional acquisition sequences for bi-component analysis. For example, a 3D cartesian turbo flash sequence has been used for data acquisition with a T1ρ preparation module consisting of a spin-lock pulse divided into four segments with an alternating phase [19,20,21]. However, there appears to be only one study on the bi-component analysis of T1ρ in the tendon, which used a pointwise encoding time reduction with radial acquisition (PETRA) sequence [32].

In this study, we aimed to evaluate the single- and bi-component relaxation of the patellar tendon ex vivo at 3T. We also investigated the relaxation times of the short and long T1ρ components and their relative fractions.

## 2. Methods

### 2.1. Specimens

Five cadaveric human knee joint specimens of average ages of 60.4 ± 12.7 years (three males, two females) were scanned. The knee specimens were provided by a nonprofit donation company (United Tissue Network, Phoenix, AZ, USA). The specimens, initially frozen at −80 °C, were thawed at room temperature for ~20 h before scanning.

### 2.2. MRI Acquisition

**Figure 1:**
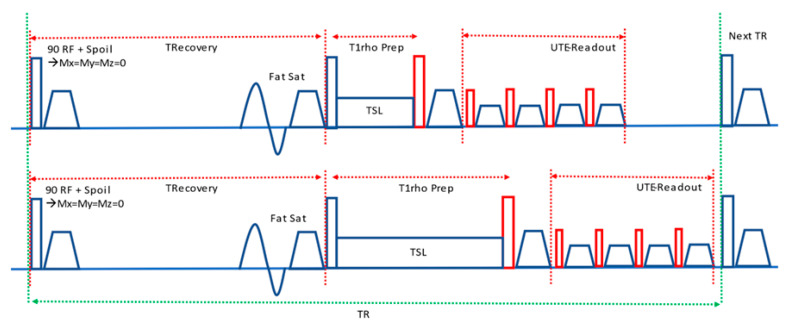
Sequence diagram. The UTE-T1ρ sequence includes magnetization reset, fat saturation, T1p preparation, and UTE data acquisition. The T1p preparation module includes two different durations of spin-locking time (TSL) (**top** vs. **bottom**).

The scans were performed on a 3T MR750 GE whole-body scanner (GE HealthCare Technologies Inc., Milwaukee, WI, USA) using an eight-channel transmit/receive knee coil. A UTE-based continuous wave T1ρ (CW-T1ρ) sequence was used for data acquisition. The pulse sequence, as shown in Figure 1, includes magnetization reset, fat saturation, T1ρ preparation, and UTE data acquisition. The magnetization reset module consists of a 90° RF pulse followed by a spoiler gradient, which ensures that the net magnetization vector is near zero (complete spoiling). Then, a chemical shift-based fat saturation pulse is employed to suppress signal from fat. T1ρ preparation is achieved by using a 90° excitation pulse to flip the longitudinal magnetization to the transverse plane, followed by a spin-lock pulse applied along the transverse magnetization vector for a duration of spin-locking time (TSL), and another 90° pulse to return the magnetization vector to the *z*-axis. Next, the UTE multi-spoke imaging sequence acquires the same k-space line twice to compensate for T1 recovery in the acquisition train with RF amplitudes (positive and negative). The data acquisition used a radial k-space trajectory.

The acquisition parameters were as follows: acquisition matrix = 256 × 256; field of view (FOV) = 16 × 16 cm^2^; slice thickness = 3 mm; number of slices = 64; sampling bandwidth = 125 kHz; TR = 584 ms; TE = 32 µs; flip angle = 60°; number of spokes = 64; magnetization recovery time = 250 ms; spin-lock amplitude = 500 Hz; and 12 different TSLs including 0.5, 2, 4, 6, 8, 10, 15, 25, 35, 45, 55, and 65 ms. The total scan time was 56 min 48 s for imaging the whole knee. Figure 1 shows the sequence diagram.

### 2.3. Single- and Bi-Exponential Analysis

**Figure 2:**
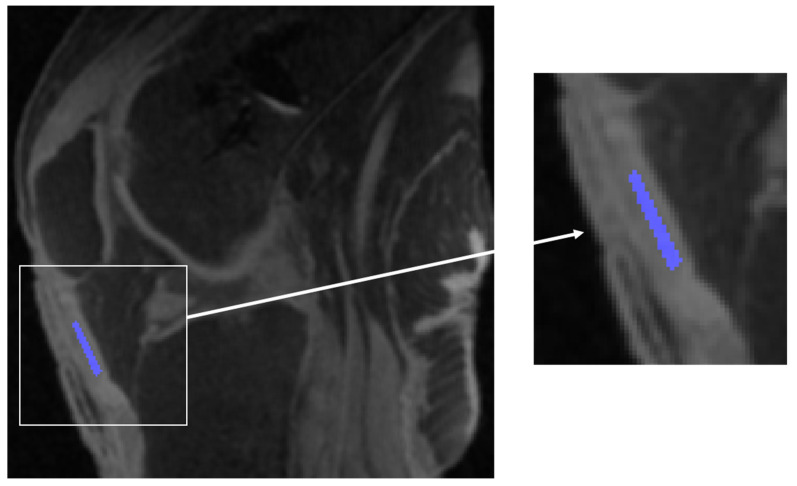
Localization of ROI (in blue) in the central region of the patellar tendon on a midsagittal slice.

A radiologist (L.S.) with seven years of experience defined the regions of interest (ROIs) in the central slices of the patellar tendon (Figure 2). The ROIs were defined in the central normal-looking region of the patellar tendon, to minimize potential partial volume effects. The single-exponential UTE-T1ρ relaxation was estimated by fitting the signal intensities of each pixel at different spin-lock durations to:

S = A × e^−TSL/T1ρ^ + N (1)

where S is the signal intensity, A is the amplitude, and N is the noise term.

The bi-exponential relaxation components were estimated using:

S = A_s_ × e^−TSL/T1ρs^ + A_l_ × e^−TSL/T1ρl^ + N (2)

where A_s_ is the amplitude of the short component and A_l_ is the amplitude of the long component. T1ρ_s_ and T1ρ_l_ are the relaxation times for the short and long components, respectively.

The noise term N is estimated by averaging the noise signals of an artifact free background region with a size of 8 × 8. Both single- and bi-exponential fitting were programmed in MATLAB version 2022a (The MathWorks Inc., Natick, MA, USA), and the Levenberg–Marquardt algorithm was used to solve the nonlinear minimization of the equations. Residual signal curves were also generated using MATLAB.

## 3. Results

**Figure 3:**
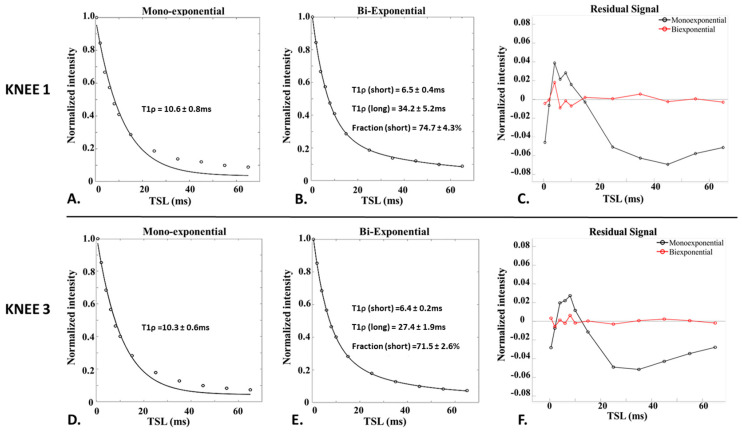
Comparison of mono-exponential (**A**,**D**) and corresponding bi-exponential fitting (**B**,**E**) and their residuals (**C**,**F**) for two knee samples. The bi-exponential model provides much improved fitting with greatly reduced residuals than the mono-exponential model.

The fitting curves for single- and bi-exponential analyses for two samples are shown in Figure 3. The bi-component signal model has superior curve fitting to the single-component model, which is evident in residual signal curves. The single-exponential fitting does not accurately represent the data as the data points deviate significantly from the fitting curve, suggesting the presence of multiple exponential components in the patellar tendon. Additionally, the bi-exponential fit exhibits very small residuals, suggesting that no more than two components are needed to accurately capture the relaxation decay. The goodness of fit measure for all bi-component model observations is above 98%, indicating excellent fit.

**Figure 4:**
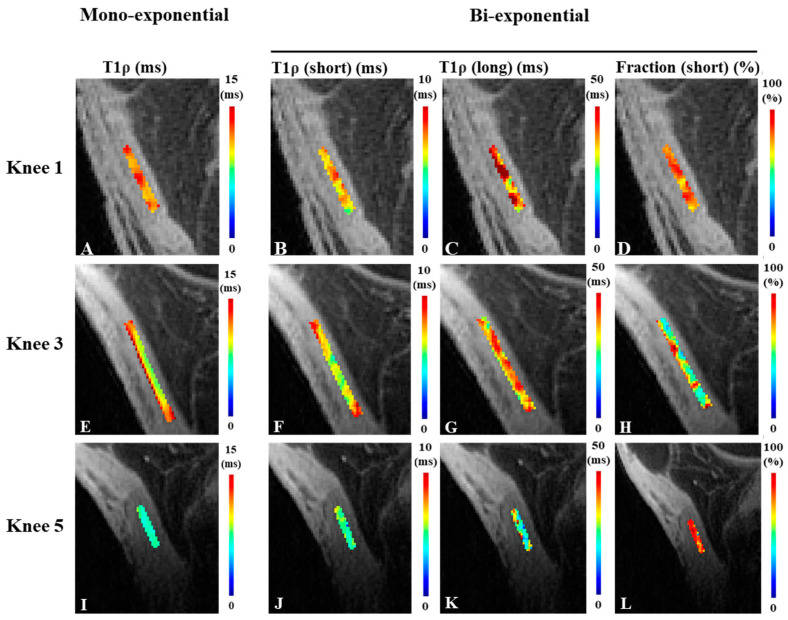
Representative T1ρ color maps in the patellar tendon of three ex vivo knees. Mono-exponential T1p relaxation maps (**A**,**E**,**I**), and bi-exponential relaxation maps of the short T1ρ component (**B**,**F**,**J**), the long T1p component (**C**,**G**,**K**), and the short component fraction (**D**,**H**,**L**).

Figure 4 shows the color maps for single- and bi-exponential analyses for three knee samples.

Table 1 shows the observed UTE-T1ρ single- and bi-exponential values for all five cadaveric human knee joints. Single-exponential UTE-T1ρ values ranged from 6.5 ± 0.3 ms to 10.6 ± 0.8 ms, with a mean value of 8.4 ± 1.7 ms. Bi-exponential analysis showed that short UTE-T1ρ values ranged from 4.6 ± 0.3 ms to 6.5 ± 0.4 ms, with a mean value of 5.5 ± 0.8 ms, and that long UTE-T1ρ values ranged from 22.7 ± 6.2 ms to 34.2 ± 5.2 ms, with a mean value of 27.4 ± 3.8 ms. The short component has a higher fraction (77.6% vs. 22.4%) than the long component. The short component fraction values across the knee samples exhibit consistency with each other, ranging from 71.5 ± 2.6% to 83.9 ± 5.7%.

## 4. Discussion

In this study, the feasibility of bi-component T1ρ analysis using UTE-MRI acquisition was examined for the first time on patellar tendons ex vivo. We were able to quantify the short and long T1ρ relaxation times using the UTE-T1ρ technique. The bi-component fit was demonstrated to be superior compared to the single-component fitting for the patellar tendon.

T1ρ relaxation time in our study was ~8.4 ms, broadly consistent with the literature. The minor variations observed among individual samples can be attributed to their distinct relaxation times, likely resulting from inherent sample variability. Du et al. [33,34] utilized a comparable CW-T1ρ sequence that involved a self-compensated spin-lock pulse for T1ρ preparation followed by 2D UTE data acquisition. They employed this sequence to investigate both in vivo and cadaveric Achilles tendon specimens. They found values ranging from 2.2 ms to 5.6 ms, which are slightly lower than in our study. This could be attributed to the different sequences for data acquisition, as the 2D UTE data acquisition used in the Du study is generally more sensitive to eddy currents than the 3D UTE data acquisition used in this study. The different tendon samples studied (the Achilles tendon in the Du study vs. the patellar tendon in this study), and the different numbers and ranges of TSLs might all contribute to the differences. Ma et al. [35] utilized CW-T1ρ preparation followed by 3D UTE Cones data acquisition, which is similar to this study. The key difference is that phase modulation was employed in this study but not in the Ma study, where T1 measurement was required to compensate longitudinal relaxation during a relatively short TR [36]. Errors in T1 compensation and different specimens might contribute to the differences in T1ρ values (~4.0 ms for the Achilles tendon vs. ~8.4 ms for the patellar tendon). Using more TSLs, as in our study, allows for the quantification of the T1ρ relaxation times with greater accuracy.

Our study found that ~77% of the relaxation component was fast-relaxing, which aligns with previous research indicating a predominantly short component in tendons [18,22,23,26,37,38,39]. Kijowski et al. [22] and Chang et al. [23] reported a ~79% fraction of short relaxation component in the patellar tendon, using UTE T2* bi-component analysis. Similarly, Liu et al. [25], Jang et al. [39], and Liu et al. [38] employed UTE T2* quantification to show that the patellar tendon had a short T2* fraction of 75–85%. Our study showed different values than Sharafi et al., who found a T1ρ short fraction of ~47% [32]. Possible reasons for this could be differences in the acquisition techniques (e.g., PETRA versus UTE), different vendor platforms, different TSLs, differences between living and postmortem tissues, and subject variability. Meanwhile, the variation in the long T1ρ component in this study is greater than that of the short component. Further research is needed to evaluate the sensitivity and specificity of bi-component UTE-T1ρ in tendon diseases.

Fat suppression is crucial for high-contrast UTE imaging of short-T2 tissues such as the patellar tendon [18]. Fat contamination and related chemical-shift artifacts may significantly affect morphological and especially quantitative UTE imaging [40]. In this study, a conventional fat-saturation module was employed to suppress fat. Chen et al. [41] demonstrated that these conventional fat saturation modules could decrease T1ρ in short T2 tissues, such as the quadriceps tendon, by approximately 9%. This reduction could be attributed to the broad frequency spectrum of these tissues, which might be in proximity to or overlapping with the signal peaks from fat. At longer spin-lock times, the efficacy of fat saturation is decreased due to the short T1 of fat, which results in partial signal recovery between two consecutive fat saturation (FS) pulses, further complicating UTE-T1ρ quantification. Moreover, magnetization transfer (MT) effects might affect the UTE-T1ρ quantification of short-T2 tissues [18]. Recently developed techniques, such as the soft–hard composite fat-saturation pulse introduced by Ma et al. [42] or the single-point Dixon approach proposed by Jang et al. [43], have the potential to improve contrast for short T2 tissues and offer more accurate measurements.

The ‘magic angle’ effect is another major confounding factor in quantitative imaging of collagen-rich tissues such as the patellar tendon [44,45,46]. Protons in water bound to collagen display dipolar interactions that are dependent on the macroscopic orientation of collagen fibers to the static magnetic field, B_0_, resulting in fast relaxation when collagen fibers are oriented at 0° to B_0_, but slow relaxation when collagen fibers are oriented at 55° to B_0_. In general, T1ρ is less susceptible to the magic angle effect than T2- and T2*-based sequences [34,47,48]. For example, Du et al. [47] found that UTE-T1ρ for the Achilles tendon increased from ~5.6 ms to ~38 ms (~6.8-fold) when the sample orientation was changed from 0° to 54° relative to the B0 field. Meanwhile, UTE-T2* increased from ~1.4 ms to ~17 ms (~12-fold) when the sample orientation was changed from 0° to 54° relative to the B0 field. A reduced magic angle effect was observed in UTE CW T1ρ [34] and UTE Adiabatic T1ρ [47,48,49]. The magic angle effect can also be suppressed by using a higher spin-lock frequency (e.g., 1–2 kHz) [49]. However, in most studies based on clinical whole-body scanners, the specific absorption ratio (SAR) limitation restricts the spin-lock frequency to 500 Hz. Moreover, at higher spin-lock frequencies, the number of measurable components is reduced [44]. Further research is needed to investigate the magic angle effect in bi-component UTE-T1ρ imaging of the patellar tendon.

One of the major limitations of this study is the long scan time for the acquisition of multiple TSLs. This could be overcome by acceleration techniques [50], reducing the number of TSLs, and acquiring fewer slices with a smaller FOV (e.g., just imaging the tendon area). Another limitation of this study is the effect of the freeze–thaw cycle on the T1ρ values. We did not investigate the bi-exponential relaxation times of the patellar tendon in vivo in this preliminary study. It is expected that both T1 and T2 relaxation times will increase with temperature [51]. However, it is unclear whether the short and long T2 relaxation times will increase with temperature at a similar rate (the water fractions remain unchanged) or at different rates (the water fractions will be different for the ex vivo study at room temperature vs. for the in vivo study at body temperature). More research is needed to investigate temperature dependence and in vivo bi-exponential behaviors. A limited number of knee samples were utilized in this study. However, this was a pilot study, implementing a relatively new quantitative MRI technique. Finally, no information on the pre-existing tendon disease and no histological correlation were available. The efficacy of 3D UTE-T1ρ bi-component analysis in the diagnosis of abnormal patellar tendon remains to be investigated.

In conclusion, the 3D UTE-T1ρ sequence was able to quantify short and long T1ρ water components in tendons and could potentially be used as a biomarker for the early detection of tendon degeneration.

## Figures and Tables

**Table 1:** Summary of mono-exponential analysis of T1ρ and bi-exponential analysis of short T1ρ, long T1ρ and short fraction values for the 5 scanned ex vivo knees.

Sample ID	Monoexponential Analysis	Bi-Exponential Analysis		
	T1ρ (ms)	T1ρ (Short) (ms)	T1 ρ (Long) (ms)	Fraction (Short) (%)
Knee 1	10.6 ± 0.8	6.5 ± 0.4	34.2 ± 5.2	74.7 ± 4.3
Knee 2	7.1 ± 0.3	5.1 ± 0.1	26.6 ± 1.9	82.5 ± 1.5
Knee 3	10.3 ± 0.6	6.4 ± 0.2	27.4 ± 1.9	71.5 ± 2.6
Knee 4	7.6 ± 0.6	4.6 ± 0.3	26.2 ± 3.4	75.2 ± 3.6
Knee 5	6.5 ± 0.3	4.9 ± 0.3	22.7 ± 6.2	83.9 ± 5.7
Mean ± SD	8.4 ± 1.7	5.5 ± 0.8	27.4 ± 3.8	77.6 ± 4.8

## Data Availability

The data presented in this study are available on request from the corresponding author.

## Abbreviations

CW	continuous wave
PETRA	pointwise encoding time reduction with radial acquisition
TE	echo time
TR	repetition time
UTE	ultrashort echo time
